# Ovariectomy Exacerbates Chronic Constriction Injury-Induced Neuropathic Pain Through Dysregulation of Spinal Estrogen Receptor Balance in Rats

**DOI:** 10.3390/ijms27219643

**Published:** 2026-10-28

**Authors:** Yu-Chen Chan, Shih-Chang Lin, Chun-Ta Huang, Ting-Yu Zheng, Ying-Chou Wang, Yi-Ju Tsai

**Affiliations:** 1 School of Medicine, College of Medicine, Fu Jen Catholic University, New Taipei City 242062, Taiwan; 2 Division of Allergy and Immunology, Department of Internal Medicine, Cathay General Hospital, Taipei City 106438, Taiwan; 3 Department of Internal Medicine, National Taiwan University Hospital, Taipei City 100225, Taiwan; 4 Department of Nutritional Science, College of Human Ecology, Fu Jen Catholic University, New Taipei City 242062, Taiwan; 5 Department of Clinical Psychology, College of Medicine, Fu Jen Catholic University, New Taipei City 242062, Taiwan

**Keywords:** estrogen receptor α, estrogen receptor β, neuropathic pain, ovariectomy, spinal cord

## Abstract

Estrogen depletion after menopause has been associated with altered pain sensitivity; however, the spinal mechanisms linking estrogen deficiency to neuropathic pain remain incompletely understood. This study investigated whether ovariectomy (OVX)-induced estrogen depletion exacerbates chronic constriction injury (CCI)-induced neuropathic pain and whether this effect is associated with changes in spinal estrogen receptor alpha (ERα) and estrogen receptor beta (ERβ) expression. Female Sprague Dawley rats underwent OVX or sham surgery, followed 5 weeks later by median nerve CCI or sham CCI. Mechanical withdrawal thresholds and thermal withdrawal latencies were evaluated using von Frey filaments and the plantar test, respectively. ERα and ERβ expression in the spinal dorsal horn was assessed via immunofluorescence staining and Western blotting. Selective ERα and ERβ agonists were administered intrathecally to examine their effects on OVX/CCI-induced pain behaviors. Plasma estradiol levels were significantly reduced after OVX, and OVX rats developed mechanical and thermal hypersensitivity. Rats in the OVX/CCI group exhibited more pronounced pain behaviors than those in the Sham/CCI group, indicating that OVX exacerbated CCI-induced neuropathic pain. In the spinal dorsal horn, ERα expression was increased in the OVX/Sham, Sham/CCI, and OVX/CCI groups, with the greatest increase observed in the OVX/CCI group. In contrast, ERβ expression was decreased in the Sham/CCI and OVX/CCI groups, with no significant difference between these groups. Intrathecal administration of an ERα agonist further aggravated mechanical allodynia and thermal hyperalgesia in OVX/CCI rats, whereas an ERβ agonist attenuated these pain behaviors. These findings suggest that OVX-induced estrogen depletion exacerbates CCI-induced neuropathic pain, potentially through a shift toward ERα-dominant signaling relative to ERβ in the spinal dorsal horn. Selective modulation of ERα and ERβ may therefore represent a potential therapeutic approach for chronic pain exacerbated by estrogen depletion.

## 1. Introduction

A substantial body of clinical [1,2] and preclinical [3,4] studies demonstrate that gonadal hormones, including estrogens, progesterone, and androgens, are important determinants of pain sensitivity and nociceptive responses. Among these hormones, estrogen has been studied most extensively. Because ovarian estrogen production declines markedly during the menopausal transition, peri- and post-menopausal women may experience a higher frequency of pain conditions, including musculoskeletal pain, headache, abdominal pain, low back pain, and fibromyalgia [5,6,7]. In parallel, sex differences in pain have received considerable research attention over recent decades [8,9,10]. Women account for a disproportionately large proportion of patients with chronic pain, including neuropathic pain [11,12,13], and may experience more severe and persistent pain symptoms than men [14]. Furthermore, anxiety and depressive disorders are more prevalent in women than in men, particularly during the peri- and post-menopause periods [15,16]. Although gonadal hormones are considered important contributors to sex-specific differences in pain, the effects of estrogen on pain modulation remain incompletely understood and, in some contexts, controversial.

Estrogen exerts its biological effects through both intracellular nuclear estrogen receptors and membrane-associated estrogen receptors [17,18]. Two classical estrogen receptor subtypes, estrogen receptor alpha (ERα) and estrogen receptor beta (ERβ), have been cloned and extensively characterized [19,20]. Morphological studies have demonstrated that both ERα and ERβ are expressed in the dorsal horn of the adult spinal cord, particularly in laminae I and II, which are critical regions for the reception and processing of nociceptive information [21,22]. Accumulating evidence suggests that ERα and ERβ have distinct, and potentially opposing, roles in the modulation of different pain states [23,24,25]. These observations raise the possibility that estrogen depletion alters spinal estrogen receptor expression and signaling, thereby modifying nociceptive processing and contributing to nerve injury-associated pain behaviors, including mechanical allodynia, thermal hyperalgesia, and spontaneous pain.

Ovariectomy (OVX) is widely used as an experimental model of ovarian hormone depletion and provides a useful approach for investigating hormone-related mechanisms relevant to peri- and post-menopausal conditions [26,27]. Chronic constriction injury (CCI), induced by loose ligation of the sciatic nerve, is a well-established model of neuropathic pain that produces robust and persistent pain-related behaviors, including mechanical allodynia and thermal hyperalgesia [28]. Regarding humankind, the majority of peripheral nerve injuries occur in the upper limbs and median nerve injury is a common form of peripheral neuropathy caused by compression, contusion, fracture-associated stretch and laceration injuries [29,30]. Therefore, we have developed an experimental model of CCI on the median nerve to induce neuropathic pain in the forelimbs of rats [31,32,33]. This modified model provides a clinically relevant experimental approach for investigating the mechanisms underlying forelimb neuropathic pain.

In the present study, we aimed to determine whether OVX-induced estrogen depletion exacerbates median nerve CCI-induced neuropathic pain behaviors in female rats. We further characterized the expression patterns of ERα and ERβ in the spinal dorsal horn following OVX and median nerve CCI. Finally, selective ERα and ERβ agonists were administered intrathecally to examine the functional roles of spinal estrogen receptors in OVX- and median nerve CCI-induced neuropathic pain.

## 2. Results

### 2.1. Ovariectomy Affects Mechanical Withdrawal Thresholds and Thermal Withdrawal Latencies

**Figure 1:**
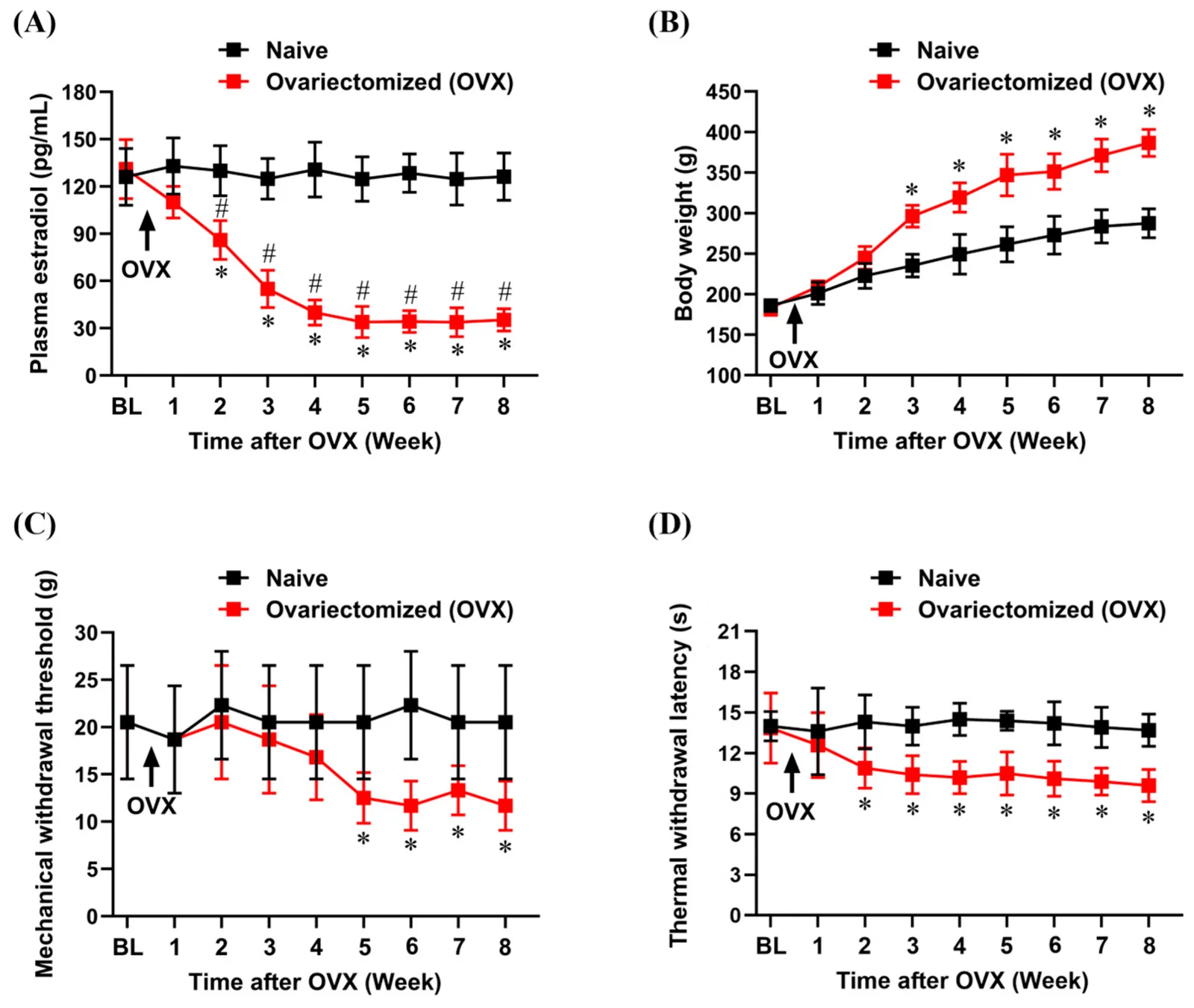
Effects of ovariectomy on plasma estradiol concentrations, body weight, and pain-related behaviors. (**A**) Plasma estradiol concentrations were comparable between naive and OVX rats at baseline. Estradiol concentrations decreased significantly after OVX and remained reduced throughout the 8-week observation period. (**B**) Body weight increased progressively in OVX rats and was significantly greater than that in naive rats from week 3 after surgery. (**C**) Mechanical withdrawal thresholds remained stable in naive rats but were significantly reduced in OVX rats from weeks 5 to 8 after surgery. (**D**) Thermal withdrawal latencies were significantly reduced in OVX rats beginning at week 2 after surgery and remained lower than those in naive rats through week 8. Data are presented as mean ± SD. *n* = 6 rats per group. # *p* < 0.05 vs. baseline by mixed-design repeated-measures analysis of variance with post hoc Bonferroni; * *p* < 0.05 vs. the naive group by mixed-design repeated-measures analysis of variance with post hoc Bonferroni. *p* for interaction < 0.05 (**A**–**D**). BL, baseline; OVX, ovariectomy.

Plasma estradiol concentrations did not differ significantly between the naive and OVX groups at baseline. However, estradiol levels were significantly reduced 2 weeks after OVX, reached low levels by week 5, and remained significantly lower than those in naive rats at all subsequent time points (Figure 1A). Accordingly, all subsequent experiments were initiated in the 5th week after OVX to ensure a stable hypoestrogenic background. OVX also resulted in a significant increase in body weight (Figure 1B). In naive rats, mechanical withdrawal thresholds assessed using von Frey filaments remained stable throughout the 8-week observation period. In contrast, OVX rats exhibited significantly reduced mechanical withdrawal thresholds from 5 to 8 weeks after surgery compared with naive rats, indicating the development of mechanical hypersensitivity (Figure 1C). Similarly, thermal withdrawal latencies in the plantar test were significantly decreased from 2 weeks after OVX and remained significantly reduced through week 8, indicating persistent thermal hypersensitivity (Figure 1D). At 8 weeks after OVX, biochemical analysis revealed that OVX rats had significantly higher plasma triglyceride and total cholesterol concentrations, but significantly lower high-density lipoprotein cholesterol concentrations, than naive rats (Table 1).

### 2.2. Ovariectomy Exacerbates CCI-Induced Neuropathic Pain

**Figure 2:**
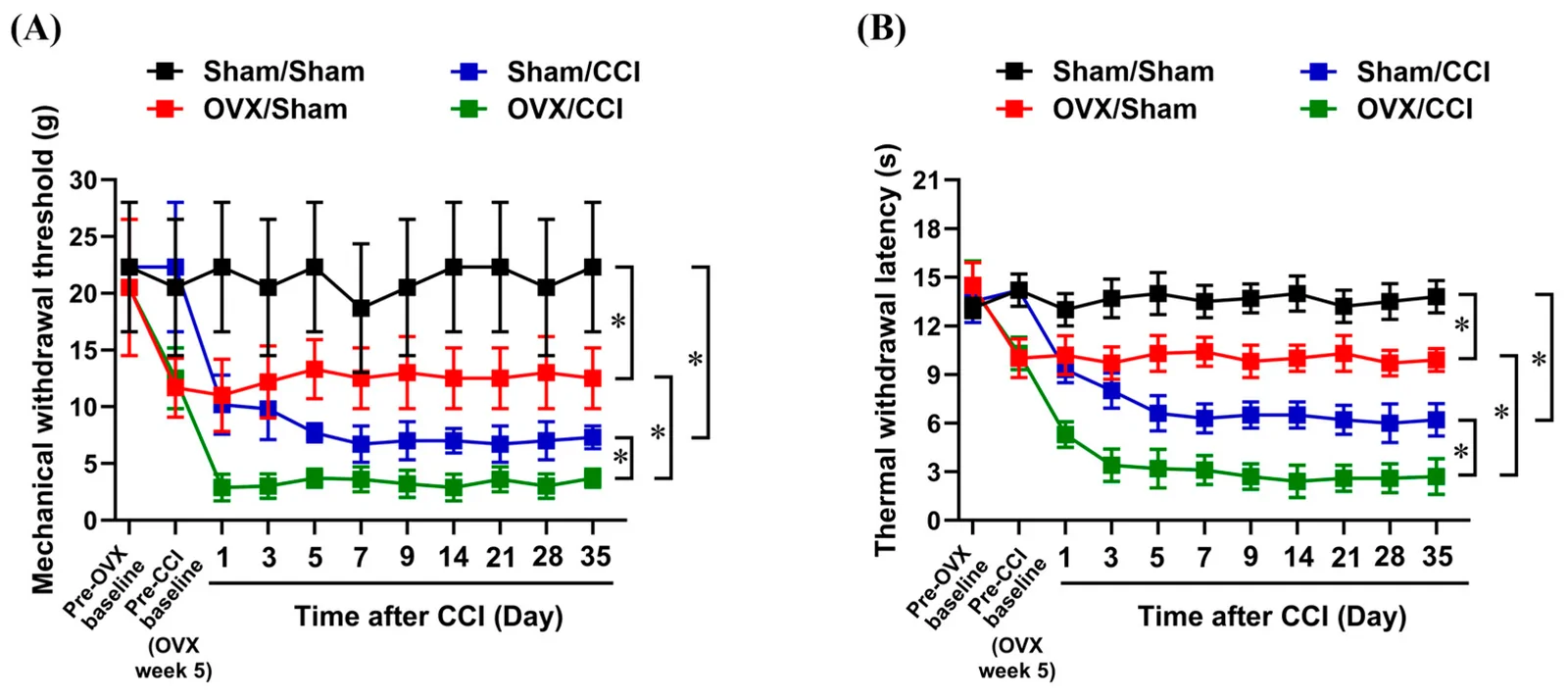
Effects of ovariectomy on CCI-induced mechanical allodynia and thermal hyperalgesia. (**A**) Mechanical withdrawal thresholds and (**B**) thermal withdrawal latencies were assessed in rats assigned to Sham/Sham, OVX/Sham, Sham/CCI, and OVX/CCI groups. CCI or sham CCI surgery was performed in the 5th week after OVX or sham OVX surgery. Following CCI, Sham/CCI rats exhibited significantly lower mechanical withdrawal thresholds and shorter thermal withdrawal latencies than Sham/Sham rats. OVX/Sham rats also showed significantly reduced mechanical withdrawal thresholds and thermal withdrawal latencies compared with Sham/Sham rats. Notably, OVX/CCI rats exhibited further reductions in both behavioral measures compared with the Sham/CCI and OVX/Sham groups, indicating that OVX exacerbated CCI-induced mechanical allodynia and thermal hyperalgesia. Data are presented as mean ± SD. *n* = 6 rats per group. * *p* < 0.05 by mixed-design repeated-measures analysis of variance with post hoc Bonferroni. *p* for interaction < 0.05 (**A**,**B**). CCI, chronic constriction injury; OVX, ovariectomy.

Mechanical withdrawal thresholds (Figure 2A) and thermal withdrawal latencies (Figure 2B) were comparable among all experimental groups at baseline. Before CCI or sham CCI surgery, no significant differences in mechanical withdrawal thresholds or thermal withdrawal latencies were observed between the Sham/Sham and Sham/CCI groups or between the OVX/Sham and OVX/CCI groups. Following CCI, rats in the Sham/CCI group exhibited significantly lower mechanical withdrawal thresholds and shorter thermal withdrawal latencies than those in the Sham/Sham group, indicating the development of mechanical allodynia and thermal hyperalgesia. Likewise, OVX/CCI rats showed significantly reduced mechanical withdrawal thresholds and thermal withdrawal latencies compared with OVX/Sham rats. Importantly, the OVX/CCI group exhibited lower mechanical withdrawal thresholds and shorter thermal withdrawal latencies than the Sham/CCI group, indicating that OVX exacerbated CCI-induced mechanical allodynia and thermal hyperalgesia. Despite these heightened pain-related responses, no self-mutilation behavior was observed in any experimental group, and no animals required early euthanasia due to neuropathic pain-related complications.

### 2.3. Ovariectomy Increases ERα, but Not ERβ, Expression in the Spinal Dorsal Horn of Rats with CCI-Induced Neuropathic Pain

**Figure 3:**
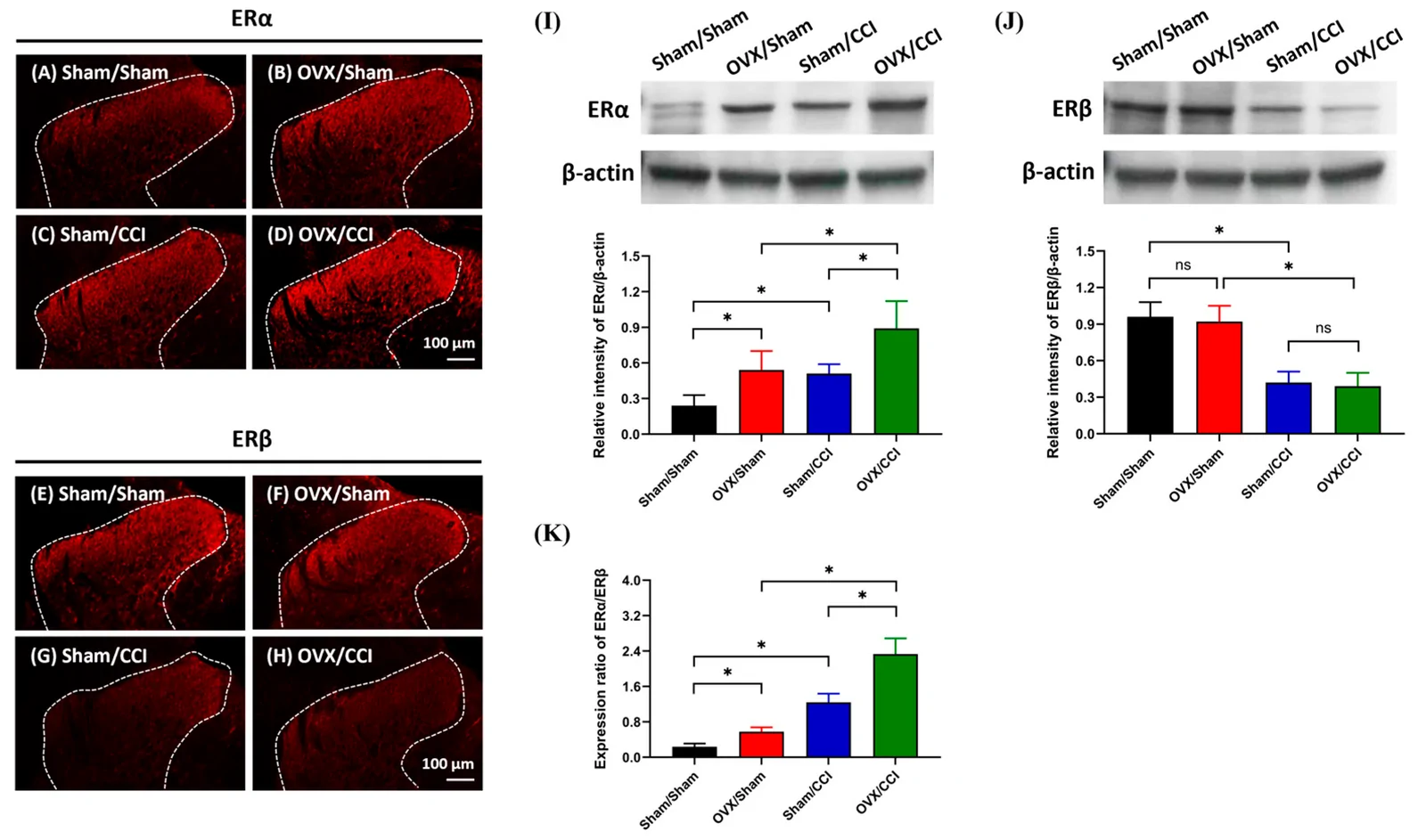
Effects of ovariectomy and chronic constriction injury on ERα and ERβ expression in the spinal dorsal horn. Representative immunofluorescence images and Western blot analyses of ERα and ERβ expression in the spinal dorsal horn were obtained 7 days after CCI or sham CCI surgery, which was performed in the 5th week after OVX or sham OVX surgery. (**A**–**D**) ERα immunoreactivity was most pronounced in the OVX/CCI group compared with Sham/Sham, OVX/Sham, and Sham/CCI groups. (**E**–**H**) ERβ immunoreactivity was reduced in the Sham/CCI and OVX/CCI groups compared with the Sham/Sham and OVX/Sham groups. (**I**) Western blot analysis showed that ERα expression was significantly increased in the OVX/Sham group compared with the Sham/Sham group and in the Sham/CCI group compared with the Sham/Sham group. ERα expression was further increased in the OVX/CCI group compared with the Sham/CCI group. (**J**) ERβ expression was significantly reduced in the Sham/CCI and OVX/CCI groups compared with the Sham/Sham and OVX/Sham groups, respectively, whereas no significant difference was observed between the Sham/CCI and OVX/CCI groups. (**K**) The ERα/ERβ expression ratio was significantly increased in the OVX/Sham, Sham/CCI, and OVX/CCI groups compared with the Sham/Sham group, with the highest ratio observed in the OVX/CCI group. Data are presented as mean ± SD. *n* = 8 rats per group. * *p* < 0.05 by two-way factorial analysis of variance. ns, not significant. *p* for interaction > 0.05 (**I**–**K**). CCI, chronic constriction injury; OVX, ovariectomy. Scale bars = 100 µm.

Representative immunofluorescence staining was used to assess ERα and ERβ expression in the spinal dorsal horn 7 days after CCI or sham CCI surgery, which was performed in the 5th week after OVX or sham OVX surgery. ERα and ERβ immunoreactivity was detected in the dorsal horn in all experimental groups. ERα immunoreactivity was markedly increased in the OVX/CCI group relative to the other experimental groups (Figure 3A–D). In contrast, ERβ immunoreactivity was reduced in OVX/CCI rats compared with Sham/Sham and OVX/Sham rats, whereas no apparent difference was observed between the OVX/CCI and Sham/CCI groups (Figure 3E–H).

Western blot analysis was subsequently performed to quantify ERα and ERβ expression in the spinal dorsal horn. ERα expression was significantly increased in the Sham/CCI and OVX/CCI groups compared with the Sham/Sham and OVX/Sham groups, respectively (Figure 3I). In addition, ERα expression was significantly higher in the OVX/Sham group than in the Sham/Sham group and was further increased in the OVX/CCI group compared with the Sham/CCI group. In contrast, ERβ expression was significantly reduced in the Sham/CCI and OVX/CCI groups compared with the Sham/Sham and OVX/Sham groups, respectively (Figure 3J). No significant difference in ERβ expression was observed between the Sham/CCI and OVX/CCI groups. To more clearly characterize the relative balance between ERα and ERβ expression, the ERα/ERβ expression ratio was compared among the experimental groups. The ERα/ERβ ratio was significantly increased in the OVX/Sham, Sham/CCI, and OVX/CCI groups compared with the Sham/Sham group, with the highest ratio observed in the OVX/CCI group (Figure 3K).

### 2.4. Intrathecal Administration of ERα and ERβ Agonists Exerts Opposite Effects on OVX/CCI-Induced Neuropathic Pain

**Figure 4:**
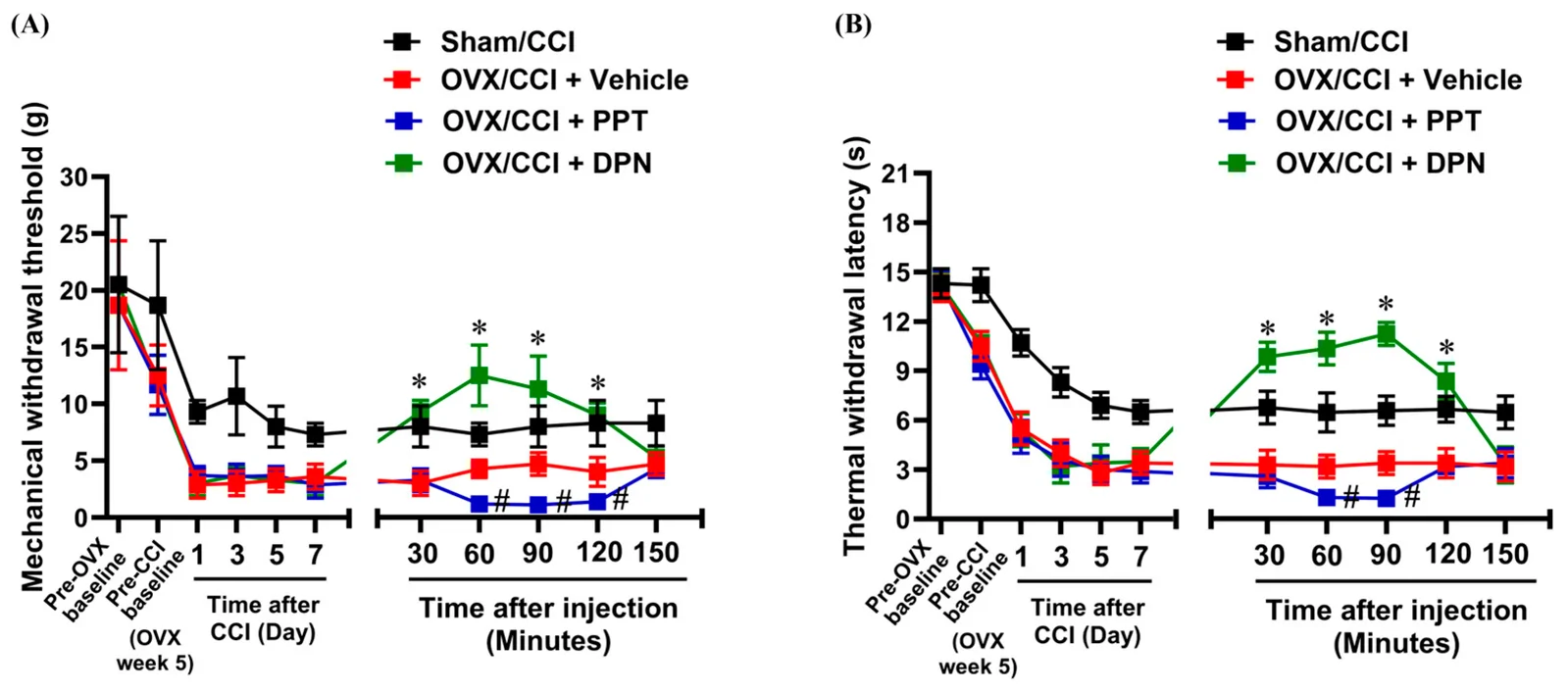
Effects of intrathecal ERα and ERβ agonists on OVX/CCI-induced mechanical allodynia and thermal hyperalgesia. (**A**) Mechanical withdrawal thresholds and (**B**) thermal withdrawal latencies were assessed in Sham/CCI rats and OVX/CCI rats treated intrathecally with vehicle, PPT, or DPN. CCI was performed on week 5 after OVX, and intrathecal treatment was administered on day 7 after CCI. Before drug administration, both mechanical withdrawal thresholds and thermal withdrawal latencies were lower in OVX/CCI rats than in Sham/CCI rats. Following intrathecal injection, the ERα agonist PPT further decreased mechanical withdrawal thresholds and thermal withdrawal latencies in OVX/CCI rats compared with vehicle-treated OVX/CCI rats. In contrast, the ERβ agonist DPN increased both measures, indicating attenuation of mechanical allodynia and thermal hyperalgesia. The effects of PPT and DPN were evident from 30 to 120 min after injection and were no longer significant at 150 min. Data are presented as mean ± SD. *n* = 6 rats per group. # *p* < 0.05 and * *p* < 0.05 vs. the OVX/CCI + vehicle group by mixed-design repeated-measures analysis of variance with post hoc Bonferroni. *p* for interaction < 0.05 (**A**,**B**). CCI, chronic constriction injury; OVX, ovariectomy.

Mechanical withdrawal thresholds (Figure 4A) and thermal withdrawal latencies (Figure 4B) were comparable among all experimental groups at baseline. Before intrathecal administration, no significant differences in mechanical withdrawal thresholds or thermal withdrawal latencies were observed among OVX/CCI rats assigned to receive vehicle, PPT, or DPN. Following intrathecal injection, PPT significantly decreased mechanical withdrawal thresholds and thermal withdrawal latencies compared with vehicle treatment, indicating that ERα activation exacerbated OVX/CCI-induced mechanical allodynia and thermal hyperalgesia. In contrast, DPN significantly increased mechanical withdrawal thresholds and thermal withdrawal latencies relative to vehicle treatment, indicating that ERβ activation attenuated OVX/CCI-induced pain hypersensitivity. These effects were most pronounced between 30 and 120 min after injection and were no longer significant at 150 min.

## 3. Discussion

This study demonstrated that mechanical withdrawal thresholds and thermal withdrawal latencies were significantly reduced beginning 5 and 2 weeks after OVX, respectively, indicating that OVX-induced estrogen depletion was associated with the development of mechanical and thermal hypersensitivity. CCI-induced mechanical allodynia and thermal hyperalgesia were more pronounced in OVX rats than in non-OVX rats. In the spinal dorsal horn, ERα expression was significantly higher, whereas ERβ expression was significantly lower, in OVX/CCI rats than in Sham/Sham rats. Furthermore, intrathecal administration of a selective ERα agonist exacerbated OVX/CCI-induced mechanical allodynia and thermal hyperalgesia, whereas administration of a selective ERβ agonist produced the opposite effect.

In the present study, plasma estradiol concentrations had decreased to low levels by 5 weeks after OVX and remained low throughout the experimental period. In addition, compared with naive rats, OVX rats exhibited significantly greater body weight, higher plasma triglyceride and total cholesterol concentrations, and lower high-density lipoprotein cholesterol concentrations. These changes are broadly consistent with metabolic alterations reported during the menopausal transition [34,35]. Collectively, these findings indicate that the OVX model employed in this study reproduced several physiological and metabolic features associated with estrogen deficiency.

Several chronic pain conditions, including migraine and temporomandibular joint pain, are more prevalent in women than in men, suggesting that estrogen contributes to pain modulation [5,7]. Preclinical studies have shown that OVX induces mechanical and thermal hypersensitivity [36,37] and enhances formalin-evoked nociceptive responses [38,39]. Consistent with these findings, the present study showed that OVX significantly reduced mechanical withdrawal thresholds and thermal withdrawal latencies, indicating the development of pain hypersensitivity following estrogen depletion. Furthermore, OVX exacerbated CCI-induced mechanical allodynia and thermal hyperalgesia. Li et al. reported that OVX alone induced mechanical and thermal hypersensitivity; however, OVX did not significantly affect sciatic nerve CCI-induced mechanical allodynia [36]. Ramer et al. reported that after cervical rhizotomy, no evidence of mechanical allodynia was found, which was contrary to the results of studies on lumbar rhizotomy [40]. This discrepancy might be attributable to regional differences in P2X3 receptor distribution between cervical and lumbar dorsal root ganglia (DRGs) [41]. These findings suggest that forelimb- and hindlimb-associated sensory pathways may differ in their nociceptive processing mechanisms and, consequently, may respond differently to estrogen deficiency. Our findings also revealed that the reduction in thermal withdrawal latency occurred earlier than the decrease in mechanical withdrawal threshold after OVX. Previous studies have indicated that estrogen negatively regulates substance P (SP) expression [42,43]. SP is predominantly expressed in small-diameter sensory neurons in the DRGs, which play an important role in thermal nociceptive transmission [44]. In addition, SP expression has been reported to fluctuate across the rat estrous cycle, supporting regulation by endogenous estrogen [45,46]. Sarajari and Oblinger further demonstrated that OVX increased SP expression in small-diameter DRG neurons, whereas estrogen replacement suppressed SP expression and produced antinociceptive effects [47]. Accordingly, we speculate that estrogen depletion may preferentially enhance SP-mediated thermal nociceptive signaling in small-diameter sensory neurons. This mechanism may contribute to the earlier onset of thermal hypersensitivity relative to mechanical hypersensitivity after OVX. However, the precise mechanism underlying OVX-exacerbated CCI-induced neuropathic pain remains unclear. Whether this exacerbation results from OVX and CCI acting on shared nociceptive pathways, thereby amplifying pain sensitization, or from the additive effects of distinct and independent mechanisms remains to be elucidated in future studies.

Estrogen regulates diverse physiological and pathological processes through estrogen receptors. In the present study, ERα expression in the spinal dorsal horn was significantly increased in the OVX/Sham, Sham/CCI, and OVX/CCI groups compared with the Sham/Sham group, with the greatest increase observed in the OVX/CCI group. In contrast, ERβ expression was significantly decreased in the Sham/CCI and OVX/CCI groups, with no significant difference between these two groups. These findings suggest that OVX-induced estrogen depletion and CCI-induced neuropathic pain are each associated with increased spinal ERα expression and that their coexistence may further enhance ERα upregulation. Conversely, the reduction in ERβ expression appeared to be associated primarily with CCI rather than OVX. Peripheral nerve injury is known to induce substantial changes in the spinal dorsal horn, including neuronal sensitization, activation of microglia and astrocytes, and increased neuroinflammatory signaling. These injury-induced changes may alter the local cellular environment and potentially influence estrogen receptor expression [23,48]. A previous study reported that ERα and ERβ colocalized with the neuronal marker NeuN, whereas no colocalization was observed with the astrocytic marker GFAP or the microglial marker OX-42 [49]. Furthermore, ERα and ERβ are expressed in interneurons within laminae I and II of the spinal dorsal horn, respectively [21,50]. Tran et al. reported that spinal ERα-positive interneurons are predominantly excitatory and may co-express SP, suggesting that increased ERα expression could facilitate nociceptive transmission [51]. Fan et al. further showed that ERβ downregulation was associated with a reduced number of interneurons and an increased density of SP-positive primary afferent fibers in lamina II [50]. This observation raises the possibility that reduced ERβ signaling may disrupt normal primary afferent–interneuron connectivity and thereby promote abnormal nociceptive signaling within the dorsal horn. Nevertheless, cell-type-specific colocalization was not performed in the present study; therefore, the cellular sources of the observed changes in ERα and ERβ expression remain uncertain. Further studies are also needed to elucidate the mechanisms underlying CCI-associated alterations in spinal dorsal horn estrogen receptor expression, particularly the potential contributions of neuroinflammatory signaling and other injury-related molecular changes.

Consistent with distinct receptor-specific roles, previous studies have shown that ERα agonist administration aggravates pain responses in OVX rats [52,53], whereas selective ERβ agonists exert antihyperalgesic effects in chemical and acute inflammatory pain models [54]. Similarly, in the present study, intrathecal administration of a selective ERα agonist exacerbated CCI-induced mechanical allodynia and thermal hyperalgesia in OVX rats, whereas ERβ agonist administration produced the opposite effect. These pharmacological findings suggest that estrogen receptor-related pathways may contribute to nociceptive modulation under estrogen-deficient conditions. Notably, the OVX/CCI group exhibited the highest ERα-to-ERβ ratio among all groups, primarily reflecting increased ERα expression rather than additional ERβ downregulation, as ERβ expression did not differ significantly between the Sham/CCI and OVX/CCI groups.

The findings of this study should be interpreted in light of limitations. First, PPT and DPN were administered only to OVX/CCI rats, without corresponding treatment groups in Sham/CCI or OVX/Sham rats. Therefore, whether their pharmacological effects are specific to the combined condition of estrogen deficiency and peripheral nerve injury remains uncertain. Second, receptor-specific antagonist blockade, dose–response analyses, and direct receptor selectivity validation were not performed. Consequently, potential receptor cross-activation or off-target effects cannot be excluded. Given these limitations, the present findings do not establish whether the observed changes in endogenous estrogen receptor expression causally contribute to the exacerbated pain hypersensitivity in OVX/CCI rats. Further studies incorporating additional experimental groups, estrogen replacement, receptor-specific antagonism, and genetic manipulation are needed to clarify the causal roles of spinal ERα and ERβ in neuropathic pain under estrogen-deficient conditions.

An additional limitation is the lack of estrous cycle monitoring in sham-operated rats during behavioral testing and at the time of tissue collection. Although Li et al. reported no significant differences in basal mechanical withdrawal thresholds or thermal tail-flick latencies across the four stages of the estrous cycle in adult female rats [36], these findings do not exclude potential effects of physiological fluctuations in ovarian hormones on CCI-induced pain hypersensitivity or spinal estrogen receptor expression. Future studies incorporating vaginal cytology and estrous cycle stage-matched comparisons would help address this potential confounding factor.

Despite these limitations, the present findings raise the possibility that selective modulation of estrogen receptor signaling may represent a potential therapeutic approach for postmenopausal chronic pain. Zhang et al. reported that long-term estrogen supplementation in OVX rats initially attenuated mechanical hyperalgesia; however, this analgesic effect gradually diminished in association with increased spinal ERα expression and decreased ERβ expression [37]. These findings suggest that, although estrogen supplementation may provide short-term analgesic benefits, prolonged treatment may be accompanied by alterations in estrogen receptor expression that reduce its therapeutic efficacy. Therefore, selective modulation of estrogen receptor signaling may represent a more targeted strategy than estrogen supplementation alone. In particular, inhibiting ERα signaling and/or enhancing ERβ signaling may help shift spinal estrogen receptor signaling toward a less pronociceptive state and alleviate chronic pain associated with postmenopausal estrogen deficiency.

## 4. Materials and Methods

### 4.1. Animals

Female Sprague Dawley rats were purchased from BioLASCO Taiwan Co., Ltd. (Taipei City, Taiwan), and weighing 170–200 g between the age of 8 and 10 weeks were used in this study. Animals were housed under standardized laboratory conditions with a 12 h light/dark cycle and had ad libitum access to food and water. All experimental procedures were designed to minimize animal pain and discomfort and were approved by the Institutional Animal Care and Use Committee of Fu Jen Catholic University (Approval No. A11429, 1 June 2025). All procedures were conducted in accordance with the ethical guidelines of the International Association for the Study of Pain.

### 4.2. Surgery

Ovariectomy (OVX) was performed under isoflurane anesthesia. Briefly, a longitudinal incision was made along the dorsal midline to expose the reproductive tract [55]. The ovaries were identified bilaterally adjacent to the Y-shaped uterine horns as pink, mulberry-like structures surrounded by adipose tissue. The oviducts were clamped with hemostatic forceps, and both ovaries were excised. The remaining tissues were gently returned to the abdominal cavity, after which the peritoneum, muscle, fascia, and skin were closed in layers using sutures. Sham-operated rats underwent the same surgical procedure, including exposure of the ovaries, but without ovarian excision. Postoperative analgesia was provided with carprofen administered subcutaneously at a dose of 5 mg/kg once daily for 7 days following OVX or sham OVX surgery.

Chronic constriction injury (CCI) of the median nerve was performed according to the method originally described by Bennett and Xie [28]. Under a dissecting microscope, the right median nerve was carefully exposed and separated from the surrounding tissues at the elbow level, between the two heads of the pronator teres muscle. Four 4-0 chromic gut ligatures were loosely tied around the nerve at approximately 1 mm intervals, and the incision was subsequently closed with sutures. Sham surgery of the right forelimb was performed using the same procedure, including exposure of the median nerve, but without placement of ligatures. No postoperative analgesics were administered following CCI or sham CCI surgery to avoid potential pharmacological interference with subsequent behavioral assessments.

Rats were randomly assigned to one of six groups: (a) naive female rats (*n* = 6); (b) ovariectomized female rats (OVX, *n* = 6); (c) sham-OVX rats subjected to sham CCI (Sham/Sham, *n* = 14); (d) OVX rats subjected to sham CCI (OVX/Sham, *n* = 14); (e) sham-OVX rats subjected to CCI (Sham/CCI, *n* = 14); and (f) OVX rats subjected to CCI (OVX/CCI, *n* = 14). CCI or sham CCI surgery was performed in the 5th week after OVX or sham OVX surgery. Rats in the naive and OVX groups were assessed for plasma estrogen concentration, body weight, pain-related behaviors, and biochemical parameters. Rats in the Sham/Sham, OVX/Sham, Sham/CCI, and OVX/CCI groups were subsequently randomly allocated to one of two experimental cohorts. In the behavioral cohort, pain-related behavioral tests were performed. In the biochemical cohort, rats were allowed to survive for 7 days after CCI or sham CCI surgery and were then processed for immunofluorescence staining and Western blot analysis.

### 4.3. Intrathecal Administration of Drugs

PPT (4,4′,4″-(4-propyl-[1H]-pyrazole-1,3,5-triyl)trisphenol), a selective ERα agonist (Tocris, Bristol, UK), and DPN (2,3-bis(4-hydroxyphenyl)propionitrile), a selective ERβ agonist (Tocris), were initially dissolved in dimethyl sulfoxide (DMSO) and subsequently diluted in artificial cerebrospinal fluid (aCSF) to a final DMSO concentration of 1%. The doses of PPT and DPN were selected according to Xu et al. [56]. PPT (1 mM; *n* = 6), DPN (5 mM; *n* = 6), or vehicle (1% DMSO; *n* = 6) was administered intrathecally using the direct transcutaneous injection technique described by Mestre et al. [57]. Briefly, rats were anesthetized with isoflurane and placed in the prone position. A 25 µL Hamilton microsyringe fitted with a 30-gauge needle was inserted between the L5 and L6 vertebrae to access the subarachnoid space. A tail flick or other transient motor response was considered evidence of successful intrathecal puncture. Drugs or vehicle were administered in a standardized volume of 10 µL. An additional control group (*n* = 6) underwent median nerve CCI but received neither OVX nor intrathecal injection. All intrathecal treatments were administered on day 7 after median nerve CCI in OVX rats. Pain-related behavioral testing was performed at 30, 60, 90, 120, and 150 min after injection.

### 4.4. Measurements of Estradiol Concentration

Rats were anesthetized with isoflurane, and blood samples were collected by tail-vein puncture into EDTA-coated tubes. Samples were allowed to stand for 1 h and were then centrifuged at 14,000× *g* for 10 min. Plasma was collected and stored at −80 °C until analysis. Plasma estradiol concentrations were measured weekly throughout the experiment using a commercially available competitive ELISA kit (QuicKey Pro Rat E2 ELISA Kit, Cat. No. E-OSEL-R0001; Elabscience Biotechnology, Houston, TX, USA) according to the manufacturer’s instructions. The assay had a sensitivity of 1.17 pg/mL and a detection range of 3.13–200 pg/mL. According to the manufacturer, no significant cross-reactivity or interference between estradiol and its analogues was observed. The manufacturer-reported intra-assay and inter-assay coefficients of variation were 3.51–6.98% and 5.40–5.89%, respectively. The mean spike recovery in EDTA plasma was 91%, and the mean dilution linearity ranged from 97% to 101%. All samples and standards were assayed in duplicate, and estradiol concentrations were calculated using a standard curve generated for each assay.

### 4.5. Behavioral Testing

#### 4.5.1. Von Frey Filament Test

Mechanical withdrawal thresholds of the forepaws were assessed using a series of von Frey filaments with bending forces ranging from 0.16 to 26.0 g (Semmes–Weinstein Monofilaments; North Coast Medical, Inc., Gilroy, CA, USA), as previously described [58]. Rats were individually placed in transparent Plexiglas chambers (25 × 40 × 18 cm) with wire-mesh floors and allowed to habituate for 30 min. The medial plantar surface of each forepaw was stimulated with von Frey filaments in ascending order of bending force [59]. Each filament was applied five times at 1 min intervals. The withdrawal threshold was defined as the lowest bending force that elicited paw withdrawal responses in at least two of the five applications.

#### 4.5.2. Plantar Test

The plantar test was performed at least 2 h after completion of the von Frey filament test. Thermal withdrawal latency of the forepaws was assessed using the plantar test, as previously described by Hargreaves et al. [60]. Rats were individually placed in transparent Plexiglas chambers (22 × 17 × 14 cm; Ugo Basile, Comerio, Italy) positioned on a 3 mm thick clear glass floor and allowed to acclimate for 30 min. A focused infrared radiant heat source was applied to the plantar surface of the forepaw, and the latency to forepaw withdrawal was recorded. To prevent tissue damage, a cutoff latency of 20 s was applied. A minimum interval of 10 min was maintained between successive measurements. Each forepaw was tested five times, and the mean withdrawal latency was calculated and rounded to the nearest 0.1 s.

### 4.6. Biochemical Measurements

Blood samples were collected from the abdominal aorta 8 weeks after OVX for biochemical analysis. Plasma concentrations of triglycerides, glucose, aspartate aminotransferase, alanine aminotransferase, total cholesterol, and high-density lipoprotein cholesterol were measured using a Cobas C111 automated analyzer (Roche, Basel, Switzerland).

### 4.7. Immunofluorescence Staining

Rats were deeply anesthetized by intraperitoneal administration of Zoletil (20–40 mg/kg) and xylazine (5–10 mg/kg), euthanized, and transcardially perfused with 0.1 M phosphate buffer (pH 7.4), followed by 4% paraformaldehyde in phosphate buffer. Spinal cord tissue blocks containing the seventh cervical segment were collected, cryoprotected overnight in 30% sucrose in phosphate buffer, and sectioned transversely at a thickness of 30 µm using a cryostat (Leica, Nussloch, Germany). Free-floating spinal cord sections were treated with 1% H_2_O_2_, blocked in 10% normal goat serum (NGS; GibcoBRL, Grand Island, NY, USA) in phosphate buffer for 1 h, and incubated for 18 h at 4 °C with either rabbit polyclonal anti-ERα antibody (1:500; Invitrogen, Waltham, MA, USA) or rabbit polyclonal anti-ERβ antibody (1:500; Invitrogen). Primary antibodies were diluted in 0.01 M phosphate-buffered saline (PBS; pH 7.4) containing 10% NGS and 0.1% Triton X-100. After washing with PBS, sections were incubated with Cy3-conjugated anti-rabbit IgG secondary antibody (1:400; Jackson ImmunoResearch, West Grove, PA, USA) for 2 h at room temperature. Sections were then mounted on gelatin-coated slides and examined under a fluorescence microscope (Leica). Negative controls were processed using the same immunofluorescence staining procedure, except that the primary antibodies were omitted.

### 4.8. Western Blot Analysis

Under deep anesthesia induced by intraperitoneal administration of Zoletil (20–40 mg/kg) and xylazine (5–10 mg/kg), rats were euthanized by decapitation. The spinal dorsal horns at the seventh cervical segment were rapidly dissected and homogenized on ice in 100 µL of lysis buffer containing 50 mM Tris-HCl (pH 8.0), 150 mM NaCl, 1 mM ethylenediaminetetraacetic acid, 1% NP-40, 0.5% deoxycholic acid, 0.1% sodium dodecyl sulfate (SDS), 1 mM sodium orthovanadate (Na_3_VO_4_), 20 µg/mL aprotinin, 20 µg/mL leupeptin, and 20 µg/mL pepstatin A. Homogenates were centrifuged at 10,000× *g* for 20 min at 4 °C, and the supernatants were collected. Total protein concentrations were determined using a detergent-compatible protein assay with bovine serum albumin as the standard (Bio-Rad, Hercules, CA, USA). Equal amounts of protein (30 µg per lane) were separated by 12% SDS–polyacrylamide gel electrophoresis and transferred onto nitrocellulose membranes (GE Healthcare, Piscataway, NJ, USA). Ponceau S staining was used after transfer to verify equal protein loading and transfer efficiency. Membranes were blocked for 1 h at room temperature in 5% nonfat dry milk prepared in phosphate-buffered saline containing 0.1% Tween-20 (PBS-T). Membranes were then incubated overnight at 4 °C with rabbit anti-ERα antibody (1:1000; Invitrogen), rabbit anti-ERβ antibody (1:1000; Invitrogen), or mouse anti-β-actin antibody (1:5000; Sigma-Aldrich, St. Louis, MO, USA). After three washes with PBS-T, membranes were incubated for 1 h at room temperature with horseradish peroxidase (HRP)-conjugated sheep anti-rabbit IgG (1:5000; GE Healthcare) or HRP-conjugated sheep anti-mouse IgG (1:5000; GE Healthcare), as appropriate. Protein bands were visualized using enhanced chemiluminescence (ECL) Western blotting detection reagents (GE Healthcare) and exposed to X-ray film for 5 min. The optical density of each immunoreactive band was quantified using Gel-Pro Analyzer software 4.0 (Media Cybernetics, Bethesda, MD, USA).

### 4.9. Statistical Analysis

Model assumptions were assessed by visual inspection of residual Q–Q plots and the Shapiro–Wilk test. Homogeneity of variance was evaluated using Levene’s test, and sphericity was assessed using Mauchly’s test. When the sphericity assumption was violated, the Greenhouse–Geisser correction was applied. There were no missing observations, and no imputation was performed.

Longitudinal outcomes were analyzed using mixed-design repeated-measures analysis of variance, with time as the within-subject factor and experimental group as the between-subject factor, including the group × time interaction. Significant interactions were followed by Bonferroni-adjusted simple-effects comparisons. Western blot outcomes were analyzed using two-way factorial analysis of variance, with OVX and CCI status as fixed factors, including the OVX × CCI interaction. ERα/ERβ ratios were natural-log transformed before analysis. Biochemical parameters were compared using the Mann–Whitney U test.

Data are presented as mean ± standard deviation, except for biochemical parameters, which are presented as median (interquartile range). A two-sided *p* < 0.05 was considered statistically significant. All analyses were conducted using SPSS for Windows, version 20.0 (SPSS Inc., Chicago, IL, USA).

## Figures and Tables

**Table 1:** Biochemical parameters in naive and ovariectomized (OVX) rats.

	Naive	OVX
Triglycerides, mg/dL	95.3 (64.5–119.7)	140.8 (121.2–187.7) *
Blood glucose, mg/dL	127.7 (97.6–148.8)	139.4 (115.7–159.4)
Aspartate aminotransferase, U/L	105.6 (70.5–128.1)	109.8 (80.3–122.7)
Alanine aminotransferase, U/L	34.2 (31.2–42.2)	45.9 (31.9–68.3)
Total cholesterol, mg/dL	88.1 (84.5–100.4)	123.8 (111.9–143.8) *
High-density lipoprotein cholesterol, mg/dL	83.7 (57.2–92.2)	46.9 (32.9–71.0) *

Values are presented as the median (interquartile range). * *p* < 0.05 as compared with the Naive group.

## Data Availability

The raw data supporting the conclusions of this article will be made available by the authors upon request.
